# The Influence of Self-Efficacy and Work Input on Physical Education Teachers’ Creative Teaching

**DOI:** 10.3389/fpsyg.2019.02856

**Published:** 2020-01-08

**Authors:** Yan Xiong, Xi-Yang Sun, Xue-Qian Liu, Ping Wang, Bing Zheng

**Affiliations:** ^1^College of Physical Education, Guangzhou University, Guangzhou, China; ^2^School of Sports Media, Guangzhou Sport University, Guangzhou, China; ^3^Department of Physical Education, Tangshan Normal University, Tangshan, China

**Keywords:** self-efficacy, work Input, creative teaching, structural model, physical edcation

## Abstract

**Objectives:**

To explore the influence of different background factors on middle school PE teachers’ self-efficacy, work input and creative teaching, and to reveal the relationship between teaching self-efficacy and work input on creative teaching.

**Methods:**

By means of self-efficacy, work engagement and creative teaching scale, a questionnaire survey was conducted among middle school PE teachers, and the data were processed and modeled by SPSS and AMOS statistical analysis software.

**Results:**

Physical education (PE) teachers’ self-teaching effectiveness was influenced by background factors such as gender, age, teaching age, full-time or part-time work and educational level. Work input was only affected by age, teaching experience and educational level, while creative teaching seemed to be only related to background factors such as educational background and full-time or part-time work; PE teachers’ general teaching effectiveness and personal teaching effectiveness had significant positive effects on energy input, concentration input, dedication input, cognitive creativity, skill creativity and emotional creativity; Concentration input had a significant positive impact on the three-dimensional of creative teaching, while energy input and dedication input had no impact on the three-dimensional of creative teaching; Work input as an intermediary variable of self-efficacy’s influence on creative teaching had been verified, but the real intermediary role was not the whole work input, but the concentration input in its structure.

**Conclusion:**

Both general and individual teaching effectiveness had positive effects on work input and creative teaching, but the energetic and dedicated input in work input cannot promote teachers’ creative teaching effectively. Therefore, the professional ethics training of PE teachers in their enthusiasm and dedication to work should be strengthened.

## Introduction

Physical education (PE) is an important part of school education. It not only provides students the opportunity to participate in sports, but also is the cradle of exploring and cultivating excellent sports talents (Zhang and Liu, 2007; Zhang, 2009). Sports experience gained in PE classes will affect the willingness of adolescence to engage in physical activities in adulthood. A PE teacher with good teaching skills, rich fitness-building knowledge and strict teaching attitude will have a profound impact on the development of students’ sports expertise and the formation of sustainable sports habits in the future (Zhang and Zhang, 2016; Behzad et al., 2018; Hannah et al., 2018). Therefore, PE teachers how to use their PE classes to perfect their own skills, put their energies into the teaching to show themselves, and to reintegrate the old teaching methods to produce more effective teaching methods, thus stimulating students’ interest in sports, cultivating students’ exercise habits, promoting students’ courage and tenacity, and promoting students’ healthy and harmonious development in physical, psychological and social adaptability will be the goals that modern PE teachers must strive for.

The theory of self-efficacy was first put forward by Bandura (Albert, 1978; Bandura, 1997) in 1977, which holds that self-efficacy refers to the individual’s confidence or belief in the ability needed to achieve his or her behavioral goals in a specific field. It affects the individual’s behavior choice, and fosters positive commitment in action because of positive self-efficacy, which determines how much effort an individual will make and how long he will persist in encountering obstacles (Eric et al., 2016; Sosan and Siamak, 2016; Sum et al., 2018). The work input was first proposed by Lodahl and Kejner (1965). The concept was mainly derived from the point of view of the psychologist Vroom (1962) on self-investment in work and the point of view of sociologist Dubin (1956) on the life interest (Dubin, 1956; Vroom, 1962; Lodahl and Kejner, 1965). Sociologists believed that work input was formed in the process of individual socialization. Therefore, work input refers to the values of general work, so it is not easy to be affected by different jobs. However, psychologists believed that work input was the degree of personal recognition of the specific work at present. Therefore, the work input will be affected by the organizational situation. Once the work changes or the organizational situation changes, it will affect the individual’s investment in the work (Chiu, 2009; Cheng, 2011). Creative teaching is not only the result of teachers’ creative thinking, but also the creativity of teachers. The comprehensive understanding of scholars at home and abroad (Zhang, 2009; Herrero et al., 2014; Ganratchakan, 2015; Rogelio and Judith, 2017) showed that the creative teaching was the teacher’s own personal creativity, and based on the students’ physical and mental development and individual differences in the teaching, using novel methods, strategies, teaching aids, media, etc. carry out the meaningful teaching activities to achieve effective teaching goals (Sternberg and Lubart, 1999; Chen and Hu, 2008; Schechter, 2012; Fan and Chang, 2013). Investigating teachers’ creative teaching needs to consider the influence of teachers’ individual and peers, school community organization, school administrative operation, school culture environment and other factors (Gardner, 2011; Jeou et al., 2016; Lisa et al., 2018).

At present, the research on self-efficacy at home and abroad mostly concentrates on two aspects: one is about the dimension of teacher self-efficacy, in order to evaluate teacher self-teaching effectiveness, and now basically forms two-way and multi-dimensional evaluation schools (Natthapol et al., 2015; Mona et al., 2018); The second is an empirical study on teachers’ self-efficacy, whose main results are that teachers with high self-efficacy will adopt more strategies to improve students’ sports performance so as to enable them to master sports skills better (Skaalvik and Skaalvik, 2010; Cristina and Andreea, 2013; Emilie et al., 2017); Teachers with high self-efficacy can carry out teaching reform and innovation in self-perception, and timely process and feedback teaching information, predict unexpected events and frequent take the initiative to check their teaching activities (David, 2009; Debra and Anthony, 2013; Andrey et al., 2016). Creative teaching is guided by certain teaching ideas, through exerting creativity and using various novel and valuable teaching strategies (Edwin, 1970; Einar and Sidsel, 2010; Yeshayahu and Sharon, 2017), in order to enhance students’ learning interest and motivation, and achieve teaching objectives. The key to creative teaching is to develop and apply novel, original or inventive teaching methods. At present, the exploration of creative teaching at home and abroad focuses on three aspects: first is the connotation of creative teaching. The creation of creativity depends on the development of creative intelligence; the performance of creativity depends on the presentation of innovative achievements (Raymond and Brian, 1987; Purcell et al., 2003). Therefore, teachers’ creative teaching, teaching innovation and innovative teaching need to be further clarified in the letter of concern Hannah et al. (2018). The second is the motivation of creative teaching. Its content mainly focuses on the intrinsic motivation, extrinsic motivation, the relationship between intrinsic motivation and extrinsic motivation of creative teaching and the evaluation of creative teaching motivation Helena and Harsha (2019). At present, relevant studies (Katherine et al., 2014; Marwa et al., 2017) have consistently shown that the higher the intrinsic motivation of teachers’ creative teaching, the more innovative performance they will have in teaching. The third is the empirical study of creative teaching. This field mainly focuses on the teaching process of teachers, to explore teachers’ beliefs, factors affecting creative teaching, and to analyze teaching strategies and evaluation methods in creative teaching (Huang and Shen, 2012; Huseyin et al., 2014; Huang et al., 2019).

To sum up, at present, the domestic and foreign research literature on PE teachers’ teaching self-efficacy, work engagement and creative teaching is relatively insufficient. Existing findings show that PE teachers’ self-efficacy affects their teaching behavior, students’ learning attitude and class management model, and there is a significant correlation between work engagement and teaching effectiveness, but the impact of work engagement and creative teaching performance is not very clear. Secondly, efficient PE teachers are often full of imagination and creativity, and can carry out teaching reform and innovation, but the relationship between self-efficacy, work engagement and creative teaching is also not very clear. Based on this, this study aims to explore the interaction among self-efficacy, work engagement and creative teaching performance of middle school PE teachers, and further reveal the differences of teachers’ self-efficacy, work engagement and creative teaching performance under different background variables, so as to provide important reference for improving the teaching efficiency of middle school PE teachers and achieving teaching objectives.

## Subjects and Methods

### Subjects

First, public middle schools in the main city of Guangzhou was sorted by number of classes, and then 20 schools were randomly selected, and all PE teachers in 20 schools were taken as the research sample. This study first adopted telephone contact with the PE section of the secondary schools to understand the number of teachers and to ask questions related to the research. Then, the investigators went to the PE teaching section of middle schools and conducted a questionnaire survey on the relevant teachers. All the questionnaires were completed and collected. A total of 415 questionnaires were sent out and 371 were recovered, with a recovery rate of 89.4%. After examination, 26 out of 371 questionnaires were found to be invalid and excluded. Reasons for exclusion: (1) those with unknown gender; (2) those who do not answer key questions. Finally, 345 valid questionnaires were obtained, and the recovery rate of valid questionnaires was 93.0%. The sample distribution was as follows: (1) Gender (male: 60.8%; female: 39.2%); Age distribution (≤35 years old: 25.6%; 36–45 years old: 38.9%; ≥46 years old: 35.5%); Marital status (married: 69.5%, unmarried:30.5%); Education level (undergraduate or below: 68.9%; master or above: 31.1%); Teaching experience (≤10 years: 24.5%; 11–20 years: 30.57%; ≥21 years: 44.93%).

### Research Methods

#### Questionnaire Design

With a structural design, it consists of five modules as follows:

(1) Basic information of subjects.

This part consisted of gender, age, marital status, educational level, teaching experience, and employment information, etc.

(2) Middle School PE Teachers Self-efficacy Scale.

Referring to Gibson and Dembo (1984) and other scholars’ bi-directional concept, teachers’ self-efficacy was constructed according to personal teaching efficacy and general teaching efficacy, which included 12 items. The concept of personal teaching effectiveness is based on teachers’ belief in their own teaching ability and skills, that is, teachers can still help students’ learning because they are limited by external unfavorable factors such as school and society, including six questions. The general concept of teaching effectiveness construction is based on teachers’ influence on students’ learning beliefs because they are limited by external factors (i.e., family, school), including six questions. The distribution of score interval of the two dimensions was 4–20 points.

Some items of this scale were as follows: (1) I can improve students’ learning motivation in a vivid way; (2) my teaching methods can improve students’ learning effect; (3) I can fully understand and master the students’ learning situation; (4) I believe that I have enough professional knowledge to solve the problem of students in learning; (5) My teaching helps students to make positive value judgments; and (6) I can change the influence of social values on students, and so on.

(3) Work engagement Scale for Middle School PE Teachers.

This scale mainly refers to Schaufeli and Bakker (2004) work engagement scale, which is constructed with three dimensions of vitality, dedication and concentration. Each dimension contains six questions, a total of 18 items. The distribution of score interval of the three dimensions is 4–20 points.

Some contents of this scale were as follows: (1) The current work is very rich, which makes me very interested; (2) I am happy to get along with the students at the current work; (3) I am willing to participate in various activities organized by the school and can cooperate with them. (4) I am willing to participate in various continuing education to enhance self-teaching effectiveness; (5) I work hard to do my job; (6) Most of my energy is spent on school work; (7) Even if there is excessive pressure, I am happy in teaching; (8) I can never complain to perform the teaching work; and (9) I can get the recognition of both students and parents in teaching, etc.

(4) Creative teaching scale for middle school PE teachers.

This scale mainly referred to the three-dimensional (cognitive, emotional, and technical) framework for construction (Liu et al., 2013). Among them, there are six items in the interaction between cognitive intention individual and environment; six items in the understanding of teachers’ willingness and creative mentality to implement creative teaching; and six items in the actual action of skilled intention teachers in creative teaching, totaling 18 items. The cognitive creative score interval was 4–20 points, and the skill creative and affective creative score range was 5–25 points.

Some contents of this scale were as follows: (1) I can be sensitive to changes in problems or things; (2) I can detect the motivating students and effectively assist them to complete creative tasks; (3) I can detect changes in teaching situations to discover or resolve problems; (4) I can independently think and judge; (5) I can design teaching units form knowledge construction through demonstration; (6) I can effectively use the most practical and creative solutions; (7) I can help students reflect on what to learn and how to learn; (8) I can design creative teaching plans; and (9) I can organize more varied and creative teaching content; and so on.

### Reliability and Validity of the Questionnaire

#### Validity Test

After four rounds of expert consultation, the scoring method of expert was adopted, that is, whether the design of each question reflects the research subject to be explored and experts were asked to make an evaluation. With the 100-point system as the standard, experts scored twice, and deleted items which average scores were less than 80 points. The average score of all items left behind was 92.3 points.

#### Questionnaire Reliability

Three middle schools in Tianhe District and Yuexiu District were selected, respectively, and their PE teachers were given the preliminary tests. The interval between the two tests was 18 days. The results showed that the questionnaire had good consistency and the correlation coefficient of test-retest was *r* = 0.84.

#### The Validity and Reliability Test of the Three Scales

The three scales were all quantified by 5-point Likert’s scale with the option of ”very disagreement, disagreement, common, consent and very consent” scored 1, 2, 3, 4, and 5 points, respectively.

Before the formal survey, the initial questionnaire were carried on the prediction test, some items which are not easy to understand were deleted, while retaining some statistics results are not ideal, but has important significance for understanding self-efficacy, work engagement and creative teaching. The mood of the items is expressed in declarative sentences (no negative sentences) and special sensitive words are avoided as far as possible. Exploratory (EFA) and confirmatory factor analysis (CFA) were used to explore the potential structure of variables (see Table 1). The structural validity and reliability of the three scales were explored separately.

**TABLE 1:** Reliability and structural validity statistics of the three scales.

**Scales**	**KMO and bartlett spherical test**	**Factor naming**	**Entry numbers**	**Explanation variance %**	**Progressive interpretation Variability %**	**Combination reliability**	**Cronbach α coefficient**
Self-efficacy							
	KMO = 0.87	Personal teaching effectiveness	4	33.67	33.67	0.87	0.81
	*P* = 0.000	General teaching effectiveness	4	25.12	58.79	0.91	0.86
Work engagement	KMO = 0.82	Vitality input	4	32.34	32.34	0.81	0.75
	*P* = 0.000	Dedicated input	4	21.15	53.49	0.83	0.79
		Devotion input	4	16.25	69.74	0.87	0.84
Creative teaching	KMO = 0.84	Cognitive creativity	4	26.69	26.69	0.86	0.82
	*P* = 0.000	Skill creativity	5	21.58	48.27	0.81	0.77
		Emotional creativity	5	17.33	65.60	0.84	0.83

It is not difficult to find from Table 1 that:

(1) The KMO values of the three subscales were 0.87, 0.82, and 0.841, respectively, and the Bartlett spherical test values reached significant levels, indicating that the three subscales were suitable for factor analysis.
(2) Two common factors can be extracted from the Instructional Self-efficacy Scale, and its cumulative contribution rate can explain 58.79% of the total variation. The load value after rotation shows that there are only four items in both common factors, that is, four items of 12 items in the initial questionnaire were deleted because of insufficient contribution. According to the content of items retained by common factors, the construction concept of individual teaching effectiveness and general teaching effectiveness is very consistent. The internal consistency Cronbach’s alpha coefficient and combination reliability of the two-dimensional degree are very high, reaching 0.81, 0.87, 0.86, and 0.91, respectively. It shows that the scale has good measurement reliability.
(3) Three common factors were extracted from the work engagement scale, and the cumulative contribution rate could explain 69.74% of the total variance. According to the load magnitude after rotation, all three common factors contained only four items, and six items in the initial scale were excluded. The contents of the items retained by the common factor are in good agreement with the construction concepts of vitality input, dedication input and devotion input. The internal consistency Cronbach’s alpha coefficient and combination reliability of the three common factors were 0.75, 0.81, 0.79, 0.83, 0.84, and 0.87, respectively. It shows that the scale has good measurement reliability.
(4) Three common factors were selected from the Creative Instruction Scale, and the cumulative contribution rate could explain 65.60% of the total variance. After rotating, the load value shows that the first common factor contains four items, the second and third common factors contain five items, and four items in the initial scale are excluded. The contents of items retained by common factors are in good agreement with the construction concepts of cognitive creativity, skill creativity and emotional creativity. The internal consistency Cronbach’s alpha coefficient and combination reliability of the three common factors were 0.82, 0.86, 0.77, 0.81, 0.83, and 0.84, respectively. It shows that the scale has good measurement reliability.

### Statistical Analysis

In this study, SPSS 19.0 and AMOS 17.0 statistical packages were used to process the survey data, and the significant level of all statistics was set at *p* < 0.05.

## Results

### Self-Efficacy Differences of Physical Education Teachers With Different Background Variables

Table 2 shows:

**TABLE 2:** The Influence of different background variables on physical education teachers’ self-efficacy.

**Variable**		**General teaching efficiency**	**Individual teaching efficiency**	**Overall teaching efficiency**
Gender	Male	3.489 ± 0.604	3.983 ± 0.581	3.738 ± 0.712
	Female	3.211 ± 0.452	3.814 ± 0.622	3.514 ± 0.506
	T	^∗^*P* < 0.05	^∗^*P* < 0.05	^∗^*P* < 0.05
Marital status	Married	3.317 ± 0.604	3.911 ± 0.428	3.614 ± 0.472
	Single	3.392 ± 0.514	3.886 ± 0.537	3.639 ± 0.556
	T	*P* > 0.05	*P* > 0.05	*P* > 0.05
Full-time or not	Yes	3.582 ± 0.602	4.121 ± 0.591	3.851 ± 0.703
	No	3.143 ± 0.472	3.675 ± 0.524	3.409 ± 0.614
	T	^∗^*P* < 0.05	^∗^*P* < 0.05	^∗^*P* < 0.05
Education level	Bachelor and below	2.777 ± 0.608	3.879 ± 1.0452	3.328 ± 0.465
	Master and above	3.931 ± 0.668	3.917 ± 1.1282	3.924 ± 0.635
	T	^∗^*P* < 0.05	*P* > 0.05	^∗^*P* < 0.05
Age	≤35 years (a)	2.982 ± 0.479	3.761 ± 0.667	3.371 ± 0.684
	36–45 years (b)	4.016 ± 0.584	3.984 ± 0.498	4.000 ± 0.416
	≥46 years (c)	3.064 ± 0.548	3.950 ± 0.713	3.507 ± 0.634
	LSD	*P*_ab_^∗^; *P*_bc_^∗^; *P*_ac_	*P*_ab_^∗^; *P*_bc_; *P*_ac_^∗^	*P*_ab_^∗^; *P*_bc_^∗^; *P*_ac_
Teaching experience	≤10 years (a)	2.939 ± 0.482	3.625 ± 0.559	3.282 ± 0.672
	11–20 years (b)	3.067 ± 0.516	3.955 ± 0.457	3.511 ± 0.721
	≥21 years (c)	4.056 ± 0.419	4.114 ± 0.631	4.085 ± 0.745
Test	LSD	*P*_ab_; *P*_bc_^∗^; *P*_ac_^∗^	*P*_ab_^∗^; *P*_bc_; *P*_ac_^∗^;	*P*_ab_^∗^; *P*_bc_^∗^; *P*_ac_^∗^

(1) From the overall level of self-efficacy, there are significant differences between male and female PE teachers (*P* < 0.05), which shows that the self-efficacy of male is higher than that of female (3.738 ± 0.712 vs. 3.514 ± 0.506); and the same rules are also observed in terms of individual teaching effectiveness and general teaching effectiveness (the scores of male to female are 3.983 ± 0.581 vs. 3.814 ± 0.622 and 3.489 ± 0.604 vs. 3.211 ± 0.452, respectively).
(2) PE teachers’ marital status had no effect on their overall self-efficacy and bidirectional degree (*P* > 0.05), but whether they belonged to full-time PE teachers had significant influence on their overall self-efficacy, general teaching effectiveness and personal teaching effectiveness (*P* < 0.05). All of them showed that full-time teachers were superior to part-time teachers (the overall score was 3.851 ± 0.703) to 3.409 ± 0.614), general and personal teaching effectiveness (*P* < 0.05). Individual scores were 3.582 ± 0.602 vs. 3.143 ± 0.472, and 4.121 ± 0.591 vs. 3.675 ± 0.524, respectively.
(3) The scores of self-efficacy and general teaching effectiveness were significantly affected by different educational level (*P* < 0.05). The scores of master degree or above were significantly better than those of undergraduate degree or below (the overall 3.924 ± 0.635 vs. 3.328 ± 0.465, and the general teaching effectiveness 3.931 ± 0.668 vs. 2.777 ± 0.608); there was no difference in personal teaching effectiveness (*P* > 0.05).
(4) Age had a significant effect on overall teaching effectiveness and both dimensions (*P* < 0.05). On the whole level, the self-efficacy of PE teachers in the 36–45 age group (4.000 ± 0.416) is significantly better than those aged 35 and below (3.371 ± 0.684) and 46 + (3.507 ± 0.634); The same pattern is observed, that is, the 36–45 age group (4.016 ± 0.584) scores the highest; the individual teaching performance is consistently expressed as 36–45 years old or older who are significantly better than those aged 35 and below.
(5) Teaching experience has significant influence on overall teaching effectiveness and bidirectional degree (*P* < 0.05). Overall, 21-year-olds (4.085 ± 0.745) are significantly better than 11-20-year-olds (3.511 ± 0.721), while the latter is significantly better than 10-year-olds (3.282 ± 0.672). General teaching effectiveness shows that 21-year-olds and older (4.056 ± 0.419) are significantly better than 20-year-olds (3.28 ± 0.672). Personal teaching effectiveness showed that those with 11 years or more were significantly better than those with 10 years or less (3.625 ± 0.559).

### Difference of Work Engagement of PE Teachers With Different Background Variables

Table 3 shows that:

**TABLE 3:** Influence of different background variables on physical education teachers’ work engagement.

**Variables**		**Vitality input**	**Dedicated input**	**Devotion input**	**Overall input**
Gender	Male	4.176 ± 0.537	4.197 ± 0.574	3.906 ± 0.482	4.156 ± 0.630
	Female	4.164 ± 0.665	4.103 ± 0.609	3.874 ± 0.629	3.984 ± 0.532
	T	*P* > 0.05	*P* > 0.05	*P* > 0.05	*P* > 0.05
Marital status	Married	4.211 ± 0.507	4.184 ± 0.634	3.815 ± 0.608	4.039 ± 0.457
	Single	4.129 ± 0.600	4.116 ± 0.509	3.965 ± 0.536	4.101 ± 0.694
	T	*P* > 0.05	*P* > 0.05	*P* > 0.05	*P* > 0.05
Full-time or not	Yes	4.019 ± 0.409	4.203 ± 0.511	3.916 ± 0.637	4.046 ± 0.714
	No	4.221 ± 0.504	4.097 ± 0.619	3.864 ± 0.563	4.094 ± 0.544
	T	*P* > 0.05	*P* > 0.05	*P* > 0.05	*P* > 0.05
Education level	Bachelor and below	3.979 ± 0.481	3.944 ± 0.664	3.661 ± 0.459	3.889 ± 0.721
	Master and above	4.361 ± 0.615	4.356 ± 0.504	4.119 ± 0.773	4.251 ± 0.706
	T	^∗^*P* > 0.05	^∗^*P* < 0.05	^∗^*P* < 0.05	^∗^*P* < 0.05
Age	≤35 years (a)	4.060 ± 0.589	4.027 ± 0.479	3.677 ± 0.566	3.993 ± 0.522
	36–45 years (b)	4.099 ± 0.501	4.094 ± 0.506	3.887 ± 0.607	3.925 ± 0.672
	≥46 years (c)	4.351 ± 0.633	4.329 ± 0.567	4.106 ± 0.409	4.292 ± 0.613
	LSD	*P*_ab_; *P*_bc_^∗^; *P*_ac_^∗^	*P*_ab_; *P*_bc_^∗^; *P*_ac_^∗^	*P*_ab_^∗^; *P*_bc_^∗^; *P*_ac_^∗^	*P*_ab_; *P*_bc_^∗^; *P*_ac_^∗^
Teaching experience	≤10 years (a)	4.054 ± 0.713	4.015 ± 0.701	3.736 ± 0.616	3.935 ± 0.721
	11–20 years (b)	4.077 ± 0.669	4.088 ± 0.744	3.742 ± 0.553	3.969 ± 0.613
	≥21 years (c)	4.381 ± 0.774	4.367 ± 0.519	4.207 ± 0.428	4.306 ± 0.515
Test	LSD	*P*_ab_; *P*_bc_^∗^; *P*_ac_^∗^	*P*_ab_; *P*_bc_^∗^; *P*_ac_^∗^	*P*_ab_; *P*_bc_^∗^; *P*_ac_^∗^	*P*_ab_; *P*_bc_^∗^; *P*_ac_^∗^

(1) The overall input score of teachers’ work is between 4.07, which indicate that the work engagement of middle school PE teachers in Guangzhou belongs to the middle and upper level. However, the three dimensions of work engagement (activity input, focus input and dedication input) and overall input have nothing to do with background factors such as gender, marriage and whether full-time teachers or not (*P* > 0.05).
(2) Different educational levels had significant effects on activity input, concentration input, dedication input and overall input (*P* < 0.05), and those with master’s degree or above were significantly better than those with bachelor’s degree or below (scores were 4.361 ± 0.615 vs. 3.979 ± 0.481, 4.356 ± 0.504 vs. 3.944 ± 0.664, 4.119 ± 0.773 vs. 3.661 ± 0.459, 4.251 ± 0.706 vs. 3.889 ± 0.721, respectively).
(3) Teachers’ age has a significant effect on overall work engagement, activity input, focus input and dedication input (*P* < 0.05). Among them, the devotion of those over 46 years old (4.106 ± 0.409) was higher than that of those over 36–45 years old (3.887 ± 0.607), while that of those under 35 years old (3.677 ± 0.566) was significantly higher than that of those under 36–45 years old (3.677 ± 0.566), while the overall work, activity and focus of those over 46 years old were significantly higher than those under 45 years old.
(4) Teacher’s working experience has a significant impact on overall work engagement and activity input, focus input and dedication input (*P* < 0.05). The consistency is that the teaching age of 21 years and above is significantly higher than that of under 21 years old.

### Differences of Creative Teaching of PE Teachers With Different Backgrounds and Variables

Table 4 shows that:

**TABLE 4:** Impact of different background factors on creative teaching of physical education teachers.

**Variables**		**Cognitive creativity**	**Skill creativity**	**Emotional creativity**	**Overall creativity**
Gender	Male	3.928 ± 0.802	3.979 ± 0.449	4.090 ± 0.706	3.862 ± 0.764
	Female	3.992 ± 0.692	3.941 ± 0.776	3.911 ± 0.503	4.058 ± 0.627
	T	*P* > 0.05	*P* > 0.05	*P* > 0.05	*P* > 0.05
Marital status	Married	3.957 ± 0.736	3.899 ± 0.747	3.932 ± 0.527	3.905 ± 0.713
	Single	3.963 ± 0.509	4.021 ± 0.573	3.988 ± 0.713	4.015 ± 0.516
	T	*P* > 0.05	*P* > 0.05	*P* > 0.05	*P* > 0.05
Full-time or not	Yes	4.114 ± 0.733	4.210 ± 0.582	4.119 ± 0.501	4.209 ± 0.7923
	No	3.806 ± 0.556	3.711 ± 0.776	3.711 ± 0.608	3.8743 ± 1.1432
	T	^∗^*P* < 0.05	^∗^*P* < 0.05	^∗^*P* < 0.05	^∗^*P* < 0.05
Education level	Bachelor or below	3.705 ± 0.682	3.731 ± 0.482	3.663 ± 0.7452	3.703 ± 0.715
	Master or above	4.215 ± 0.702	4.189 ± 0.671	4.257 ± 0.625	4.217 ± 0.802
	T	^∗^*P* < 0.05	^∗^*P* < 0.05	^∗^*P* < 0.05	^∗^*P* < 0.05
Age	≤35 years (a)	3.945 ± 0.547	3.825 ± 0.472	3.906 ± 0.634	3.953 ± 0.521
	36–45 years (b)	4.010 ± 0.501	3.959 ± 0.501	4.011 ± 0.530	3.991 ± 0.635
	≥46 years (c)	3.925 ± 0.751	4.096 ± 0.662	3.963 ± 0.506	4.017 ± 0.784
	LSD	*P*_ab_; *P*_bc_; *P*_ac_	*P*_ab_; *P*_bc_; *P*_ac_	*P*_ab_; *P*_bc_; *P*_ac_	*P*_ab_; *P*_bc_; *P*_ac_
Teaching experience	≤10 years (a)	3.909 ± 0.651	3.974 ± 0.509	3.899 ± 0.617	4.054 ± 0.703
	11–20 years (b)	3.974 ± 0.527	3.906 ± 0.773	3.992 ± 0.563	3.911 ± 0.531
	≥21 years (c)	3.997 ± 0.603	4.000 ± 0.632	3.982 ± 0.668	3.915 ± 0.627
Test	LSD	*P*_ab_; *P*_bc_; *P*_ac_	*P*_ab_; *P*_bc_; *P*_ac_	*P*_ab_; *P*_bc_; *P*_ac_	*P*_ab_; *P*_bc_; *P*_ac_

(1) The average score of creative teaching is more than 4.0, which means that the creative teaching level of PE in the main city of Guangzhou is higher. Moreover, the overall creativity and three-dimensional creativity teaching (cognition, skills and affection) have nothing to do with background factors such as gender, marriage, age and teaching age (*P* > 0.05).
(2) Different educational levels had significant effects on cognition, skills, affection and overall creativity (*P* < 0.05), and those with master’s degree or above were significantly better than those with bachelor’s degree or below (the scores were 4.215 ± 0.702 vs. 3.705 ± 0.682, 4.189 ± 0.671 vs. 3.731 ± 0.482, 4.257 ± 0.625 vs. 3.663 ± 0.745, 4.217 ± 0.802 vs. 3.703 ± 0.715, respectively).
(3) Whether they belong to full-time PE teachers has significant influence on cognition, skills, affection and overall creativity (*P* < 0.05), and the consistent performance is that full-time teachers are significantly better than part-time teachers (scores are 4.114 ± 0.733 vs. 3.806 ± 0.556, 4.210 ± 0.582 vs. 3.711 ± 0.776, 4.119 ± 0.501 vs. 3.711 ± 0.608, 4.209 ± 0.792 vs. 3.8743 ± 1.14).

### Impact of PE Teachers’ Self-Efficacy and Work Involvement on Creative Teaching Performance

**FIGURE 1:**
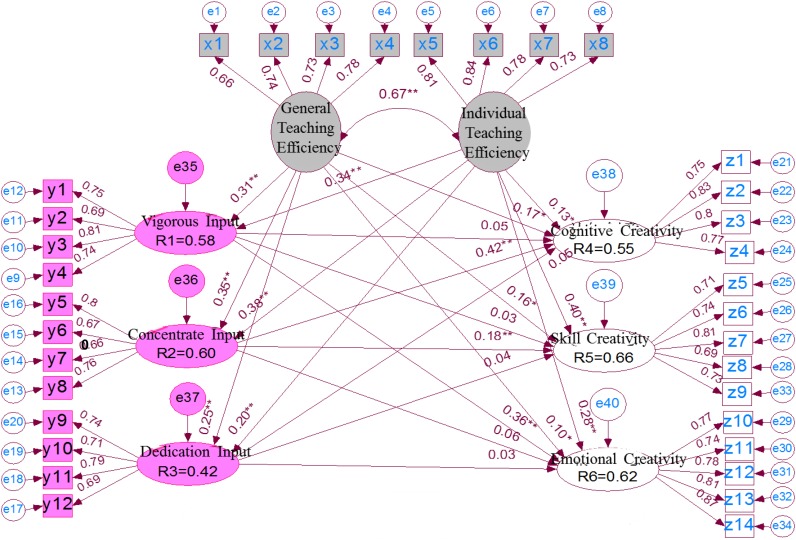
Structural model of the impact of teaching efficiency and work engagement on creative teaching.

Table 5 and Figure 1 show that:

**TABLE 5:** Structural goodness-of-fit test for global sample model.

**Causal relationship**	**Path coefficient β**	**Significant level and test results**	**Causal relationship**	**Path coefficient β**	**Significance level and test results**
Vigorous input ← motivation of participation in general teaching effectiveness	0.31^∗∗^	*P* = 0.00 < 0.01 support	Emotional input ← individual teaching efficiency	0.28^∗∗^	*P* = 0.00 < 0.01 support
Vigorous input ← general teaching efficiency	0.35^∗∗^	*P* = 0.00 < 0.01 support	Cognitive creativity ← vitality input	0.05	*P* = 0.58 > 0.05 non-support
Dedication input ← general teaching efficiency	0.25^∗∗^	*P* = 0.00 < 0.01 support	Skill creativity ← vitality input	0.03	*P* = 0.19 > 0.05 non-support
Cognitive creativity ← general teaching efficiency	0.17^∗^	*P* = 0.034 < 0.05 support	Emotional creativity ← vigorous input	0.06	*P* = 0.52 > 0.05 non-support
Skill creativity ← general teaching efficiency	0.16^∗^	*P* = 0.044 < 0.05 support	Cognitive creativity ← concentrate input	0.42^∗∗^	*P* = 0.00 < 0.01 support
Emotional creativity ← general teaching efficiency	0.36^∗∗^	*P* = 0.00 < 0.01 support	Skill creativity ← concentrate input	0.18^∗∗^	*P* = 0.00 < 0.01 support
Vigorous input ← individual teaching efficiency	0.34^∗∗^	*P* = 0.00 < 0.01 support	Emotional creativity ← concentration input	0.06	*P* = 0.087 > 0.05 non-support
Concentrate input ← individual teaching effectiveness	0.38^∗∗^	*P* = 0.00 < 0.01 support	Cognitive creativity ← dedication input	0.05	*P* = 0.17 > 0.05 non-support
Dedication input ← individual teaching effectiveness	0.20^∗∗^	*P* = 0.00 < 0.01 support	Skills creativity ← dedication input	0.04	*P* = 0.32 > 0.05 non-support
Cognitive creativity ← individual teaching efficiency	0.13^∗^	*P* = 0.029 < 0.05 support	Emotional creativity ← dedication input	0.03	*P* = 0.13 > 0.05 non-support
Skill input ← individual teaching effectiveness	0.40^∗∗^	*P* = 0.00 < 0.01 support			

*Chi square value *X*^2^,*X*^2^/df = 1.09;43.8; *P* = 0.24; GFI = 0.948 (≥0.9), AGFI = 0.951 (≥0.9), RMSEA = 0.026 (<0.1), RMR = 0.039 (<0.1), NFI = 0.937 (≥0.9), CFI = 0.974 (≥0.9), RFI = 0.916 (≥0.9), IFI = 0.924 (≥0.9), NNFI = 0.955 (≥0.9), PGFI = 0.611 (≥0.5), PNFI = 0.708 (≥0.5).*

(1) In the basic Goodness-of-fit Test of the model, the chi-square value *X*^2^/df = 1.09, which is between 1 and 3; *P* = 0.24 > 0.05, which means that the model fits well; (2) From the overall fit point of view, the goodness-of-fit index (GFI) and the adjusted goodness-of-fit index (AGFI) must be greater than 0.90, the results of this study are GFI = 0.948, AGFI = 0.951, which shows that the model fits fairly well. The RMSEA must be less than 0.1. The RMSEA of this study is 0.026. It shows that the revised model can properly explain the relationship between self-efficacy, work engagement and creative teaching. In addition, benchmark fit index (NFI) and comparative fit index (CFI), when the value is greater than 0.90, the model fitness is better, and when the value is closer to 1, the model fitness is better. In this study, NFI = 0.937, CFI = 0.974, which means that the overall fit of this study is better. The chi-square ratio is 1.09, ranging from 1 to 3. By synthesizing all the pointer judgments, it shows that the overall matching of the structural model in this study is acceptable.
(2) Standardized path coefficients in structural models represent the strength and direction of the relationship between variables, and the test of path coefficients should be significant. The interpretation ability of the model can be judged by the value of *R*_2_. From the path coefficients and the value of *R*_2_ among potential variables, the adaptability of the structural model and empirical data can be shown. The higher the *R*_2_ value, the better the mode interpretation power. Therefore, *R*_2_ is the basis for judging the explanatory ability of the model.

It is not difficult to see from Figure 1 that teachers’ general teaching effectiveness has significant positive effects on their energy input, concentration input and dedication input, with path coefficients of 0.31^∗∗^, 0.35^∗∗^, 0.25^∗∗^, respectively; teachers’ personal teaching effectiveness also has significant positive effects on energy input, concentration input and dedication input, with path system beta of 0.34^∗∗^, 0.38^∗∗^, 0.20^∗∗^, respectively. Teachers’ general teaching effectiveness has a significant positive impact on cognitive creativity, skill creativity and emotional creativity. The path coefficient beta is 0.17^∗^, 0.16^∗^, 0.36^∗∗^. Teachers’ personal teaching effectiveness has a significant positive impact on cognitive creativity, skill creativity and emotional creativity, with path coefficients of 0.13^∗^, 0.40^∗∗^, and 0.28^∗∗^, respectively. The vitality input of teachers’ work involvement has no significant impact on cognitive creativity, skill creativity and emotional creativity. The path coefficients are 0.05, 0.03, and 0.06, respectively, corresponding to *P* > 0.05. This means that energy input has no effect on teachers’ creative teaching performance. Teachers’ devotion to work has a significant positive effect on cognitive creativity and skill creativity. The path coefficient beta is 0.42^∗∗^ and 0.18^∗∗^, respectively, but it has no effect on emotional creativity, beta = 0.06, *P* > 0.05. Teachers’ devotion to work has no significant effect on cognitive creativity, skill creativity and emotional creativity. Path coefficients beta are 0.05, 0.04, and 0.03, respectively.

(3) The two dimensions of teachers’ self-efficacy, general teaching effectiveness and personal teaching effectiveness, can explain about 33.64% of the variance of vigor input (*R*_1_ = 0.58, *R*_1_^2^ = 0.3364), 36% of the variance of focus input (*R*_2_ = 0.60, *R*_2_^2^ = 0.3600) and 17.64% of the variance of dedication input (*R*_3_ = 0.42, *R*_3_^2^ = 0.1764), that is, the variance of concentration input is the highest. The two dimensions of general teaching effectiveness and individual teaching effectiveness, as well as the three sub-dimensions of energy input, concentration input and dedication input, can explain about 30.25% of the variance of teachers’ creative teaching performance (*R*_4_ = 0.55, *R*_4_^2^ = 0.3025), 43.56% of the variance of skills creative variables (*R*_5_ = 0.66, *R*_5_^2^ = 0.4356) and emotional creative variables (*R*_6_ = 0.62, *R*_6_^2^ = 0.3844), and about 38.44% of the variance, among which the ability of explaining skills and creativity variables is the highest.

## Discussion

### Self-Efficacy Characteristics of Physical Education Teachers From Different Background Variables

This study found that besides marital status, teachers’ gender, age, educational background and full-time or part-time background factors have significant effects on self-efficacy. Previous study (Khurshid et al., 2012) reported that the female middle school teachers’ self-efficacy was higher than that of men; however, some other findings pointed out that male PE teachers’ self-efficacy was higher than that of female teachers (Ozkal, 2014; Yalçin incik and Kiliç, 2014); These showed that there was difference in self-efficacy between male and female teachers. The results of this study were basically consistent with the findings of domestic scholars (Ho, 2009), but contradicted with those of foreign scholars (Esma, 2012; Shahnaz et al., 2013; Sara and Daniela, 2016). The reasons need to be further explored. In the research and exploration of domestic scholars, Lin and Qiu (2008) found that the self-efficacy of middle school PE teachers has nothing to do with age, but the self-efficacy of those who have been teaching for 16 years or more was significantly higher than that of those who have been teaching for less than 10 years; This study was consistent with their findings. In terms of whether full-time or part-time, the conclusions of this study support the viewpoint of many scholors that full-time teachers have better self-efficacy (Chen, 2010; Khurshid et al., 2012; Ozkal, 2014; Robert et al., 2018). This seemed that the full-time teachers have more contact with students and know more about students’ habits, so they have confidence in themselves, thus indirectly improving their sense of self-efficacy.

### Work Engagement Characteristics of PE From Different Background Variables

This study found that the work engagement of middle school PE teachers was not related to their gender, marital status and whether they were full-time or not, but their educational background, teaching age and age significantly affected their work engagement. In terms of gender factors, Fryer and Collings (1991) reported that male teachers devote more attention to their work than female teachers; studies by Ozkal (2014) showed that there was no gender difference in middle school PE teachers’ work engagement. In terms of marital status, some studies pointed out that marital status affected teachers’ work engagement (Marita, 2013); Study by Zhang (2013) showed that married PE teachers’ overall input and dedication were significantly higher than unmarried PE teachers. From the age structure, Robert et al. (2018) found that the work engagement of middle school PE teachers aged 41 to 50 was significantly higher than that of those under 30. From the educational level, Mona et al. (2018) found that the work engagement of P. E. teachers with master’s degree or above was significantly higher than that of those with bachelor’s degree or below. From the point of view of full-time or not, some studies (Chen, 2006; Yan, 2011; Bonnie and Hannah, 2016; Zhou et al., 2018) pointed out that the transfer of PE teachers can focus more on teaching work, and thus have higher work engagement than the concurrent (Song, 2011; Lynn et al., 2013; Zhang, 2013). From the perspective of teaching age structure, Dai and other studies show that the work engagement of those who have been teaching for more than 16 years is significantly lower than that of those who have been teaching for less than 16 years (Zhang and Zhang, 2016). It can be seen that this study is in good agreement with many scholars in terms of age, teaching age and educational background. The reason may be that for PE teachers with relatively long ages and teaching ages, they are less affected by family factors, so they can spend more time in their work. As for their academic qualifications, those with master’s degree or above will focus more on their work engagement. Is this related to the graduate study they have experienced? This is a topic worthy of further discussion. This study has great variability with related research reports on gender, marital status and whether full-time or not, and more studies are needed to verify the more contradictory variables.

### Creative Teaching Characteristics of PE Teachers From Different Background Variables

This study found that creative teaching is only affected by educational background and full-time factors, and has nothing to do with the gender, marital status, age and teaching age of middle school PE teachers. Fryer and Collings (1991) reported that teachers’ creative teaching was obviously influenced by gender factors; however, Ozkal’s (2014) research found that teachers’ creative teaching had nothing to do with gender; domestic scholar Zhang and Zhang (2016) research found that creative teaching had nothing to do with gender, while Wu’s (2011) research showed that female teachers were better than male teachers in the implementation of teaching innovation. It can be seen that whether gender factors affect creative teaching may be related to social expectations, gender equality and social changes, and the impact of gender factors should be further explored. From the perspective of teaching age structure, study by Liu et al. (2013) showed that teachers of 16–25 years’ teaching age had the best performance in creative teaching, while Lin et al.’s (2009) research results show that the teachers with shorter teaching age have better creative teaching. From the educational level, Mona et al. (2018) research points out that the overall innovation ability and teaching innovation of postgraduate and above teachers are higher than those of undergraduate and below teachers, and Natthapol et al. (2015) has the same findings, which is consistent with the conclusions obtained in this study. From the perspective of full-time teachers, the conclusions of this study are consistent with many scholars. For example, Hong believes that full-time teachers are better than part-time teachers in teaching creativity, cognitive creativity and skill creativity, and many other studies have the same results (Song, 2011; Lynn et al., 2013; Zhang, 2013). The reason may be that full-time teachers can accumulate academic and professional fields and teaching experience, and they are more able to think about creative teaching methods, so part-time teachers have better performance in creative teaching. Further interviews found that there are more administrative workers among part-time teachers, which may be that part-time administrative work consumes more time and energy of teachers, resulting in their exhaustion and lack of energy to seek new changes in teaching.

### The Influence of PE Teachers’ Self-Efficacy and Work Engagement on Creative Teaching

(1) Structural equation model shows that there is a significant positive correlation between teachers’ self-efficacy and work engagement, which is basically consistent with the findings of Yang et al. (2006) and similar to those of Ereno and Nunez (2014). Further analysis shows that general teaching effectiveness and personal teaching effectiveness have significant positive effects on the three-dimensional work engagement (energy input, focus input, and dedication input). Therefore, teachers hope that if they can improve the general teaching efficiency and master the difficulty of teaching work (personal teaching effectiveness), then they must have more energetic input, dedication and dedication. This result greatly supports some previous studies, that is, teachers with high self-efficacy will show more active teaching behavior and help others more actively (Cristina and Andreea, 2013; Emilie et al., 2017). Lin et al. (2009) pointed out that teacher with high self-efficacy will fully plan and prepare, adjust and evaluate their teaching behavior and effectiveness after class, so self-efficacy plays a decisive role in teachers’ teaching self-monitoring ability.
(2) Structural equation model shows that among the three dimensions of work engagement, vitality input, focus input and dedication input, only focus input has a significant positive impact on cognitive creativity, skill creativity and emotional creativity in teachers’ creative teaching performance, which confirms the findings of Kalpana and Shibu (2014) and other scholars. Previous studies have shown that the degree of teachers’ work involvement will affect the performance of teaching effectiveness, that is, high work involvement is conducive to Teacher-Student interaction, thus forming a positive teaching effectiveness (Martin, 2001; Behzad et al., 2018; Hannah et al., 2018). However, previous studies have not explored the impact of job involvement on creative teaching in terms of its sub-dimensions. Therefore, it is only found that as long as more job involvement is involved, teachers’ creative teaching performance can be positively improved. This study was surprised to find that energy input and dedication have little effect on improving teachers’ creative teaching performance. Only when teachers concentrate on their work, feel proud of their own work, immerse themselves in their work and feel happy when they work conscientiously, can they positively affect teachers’ cognitive creativity, skill creativity and emotional creativity. However, there is no significant influence on teachers’ creative teaching performance in the performance of vigorous input, such as being energetic in their work, feeling energetic in their dedication, feeling satisfied with their life conditions, and thinking that all aspects of life are close to their ideal. This is a result not found in previous studies, and it is worth further exploring its stability in the future. From the model standardization path coefficient (beta), teachers’ dedication to cognitive creative teaching behavior showed the greatest performance (beta^∗∗^ = 0.42), while emotional creative teaching behavior showed the smallest performance (beta = 0.06). Therefore, in the expression of emotional creative teaching behavior, how to create some novel teaching environment atmosphere needs to invest more enthusiasm and thoughts.
(3) Structural equation model shows that teachers’ self-efficacy has a significant positive impact on teachers’ creative teaching, which is consistent with Ozkal’s research results. From the perspective of two-way self-efficacy, general teaching effectiveness and personal teaching effectiveness have significant positive effects on teachers’ cognitive creativity, skill creativity and emotional creativity in creative teaching performance, which supports previous research findings of Wu (2011). Tierney and Farmer (2002) and other studies have found that whether teachers have a high sense of teaching self-efficacy is the key factor affecting whether teachers dare to innovate and show creative teaching in the classroom. Study by Wu (2011) also points out that the key point of creative teaching is that in the process of teaching, teachers integrate their own creativity into teaching behavior, making their activities diversified and lively. The main purpose of teaching is to achieve teaching objectives through creative teaching methods. It can be seen that creative teaching has positive and positive significance and value for students’ learning. From the influence coefficient, it can be seen that teachers’ personal teaching effectiveness has a higher influence on skills creativity (beta^∗∗^ = 0.40) and emotional creativity (beta^∗∗^ = 0.28). Therefore, teachers’ personal teaching effectiveness can be chosen as the priority.

## Conclusion

(1) There are many factors that influence the self-efficacy of PE teachers except marital status, such as gender, age, teaching age, full-time or part-time employment and educational level. Job involvement is only affected by age, teaching age and educational level, which is independent of background factors such as gender, marital status and full-time or part-time employment. Creative teaching has the least influence. Creative teaching has the least influence, which is only relating to academic qualifications, full-time or part-time employment, etc., and it has nothing to do with teacher’s gender, marital status, age and teaching age.
(2) General teaching effectiveness and individual teaching effectiveness of PE teachers have positive influence on job involvement in three aspects, such as energy input, concentration input and dedication input. General teaching effectiveness and individual teaching effectiveness also have positive effects on three aspects of creative teaching, such as cognitive creativity, skill creativity and affective creativity.
(3) In the three dimensions of teacher work engagement, only the focus on input has a significant influence on the three facets of teachers’ creative teaching performance, while the vitality input and contribution input have no positive influence on the three dimension of teachers’ creative teaching performance; As an intermediary variable of self-efficacy’s influence on creative teaching, PE teachers’ work engagement has been verified, but this intermediary force is not the whole work engagement, but one of its three aspects.

## Limitations

(1) This study found that PE teachers’ gender, age, marital status, full-time or part-time, educational level, teaching age and other background information affected their self-efficacy, work input and creative teaching to some extent, but there were many inconsistencies in their findings by domestic and foreign scholars. These differences may be related to the different sample size distribution and variable processing methods adopted by different scholars. For example, in this study, the male sample size was about twice that of the females, and the married sample size was about twice that of unmarried women. The teaching age was ≤10 years as a category, and this may ignore the fact that the teaching experience of teachers with a teaching age of ≤5 years was seriously insufficient. It is suggested that the future research should standardize the background information of the subjects, so as to more accurately reveal the relationship between these variables.
(2) This study found that the general teaching effectiveness and individual teaching effectiveness of PE teachers had a significant positive impact on the work input and creative teaching performance, but the influence of teachers’ energy input and dedication input on creative teaching had not reached a significant level. This seemed to remind us that after the teachers’ teaching skills and educational level were upgraded, they can effectively influence the work input and creative teaching performance. If more energy and dedication were put in, they will have the opportunity to provide the students with a warm learning environment and reliable spiritual support, but these speculations had yet to be further explored in the future.

## Data Availability Statement

The raw data supporting the conclusions of this article will be made available by the authors, without undue reservation, to any qualified researcher.

## Ethics Statement

Ethical review and approval were not required for the study on human participants in accordance with the local legislation and institutional requirements. The patients/participants provided their written informed consent to participate in this study.

## Author Contributions

All authors designed the study, contributed, and approved the final manuscript. YX, PW, and X-QL performed the questionnaire survey. YX and X-YS wrote the manuscript. BZ undertook the statistical analysis.

## Conflict of Interest

The authors declare that the research was conducted in the absence of any commercial or financial relationships that could be construed as a potential conflict of interest.
